# “Celts” up and down the Alps. Insights on mobility patterns in the pre‐Roman/Celtic population from Verona (NE Italy, 3rd–1st c. BCE): A multi‐isotopic approach

**DOI:** 10.1002/ajpa.24523

**Published:** 2022-04-26

**Authors:** Zita Laffranchi, Arsenio Granados‐Torres, Sandra Lösch, Albert Zink, Irene Dori, Antonio Delgado‐Huertas, Marco Milella

**Affiliations:** ^1^ Department of Physical Anthropology, Institute of Forensic Medicine University of Bern Bern Switzerland; ^2^ Stable Isotopes Biogeochemistry Laboratory Andalusian Institute of Earth Sciences, (IACT‐CSIC‐UGR) Granada Spain; ^3^ Institute for Mummy Studies Eurac Research Bolzano Italy; ^4^ Soprintendenza Archeologia Belle Arti e Paesaggio per le province di Verona Verona Italy

**Keywords:** Alps, Celtic populations, late iron age, mobility patterns, stable isotopes

## Abstract

**Objectives:**

The Late Iron Age in continental Europe featured complex demographic processes including, among others, the establishment of transalpine “Celtic” communities on the Italian peninsula between the 4th and 1st centuries BCE. To date, only few data are available about mobility and migration in these populations. Here we explore these topics among the Cenomani of Seminario Vescovile (SV‐Verona, Italy, 3rd–1st c. BCE) through a multi‐isotopic approach and test the possible associations with sex, age and funerary treatment.

**Materials and methods:**

We analyzed isotopic ratios of oxygen (*δ* ^18^O) and carbon (*δ* ^13^C) from bone phosphate and collagen, respectively, of 49 individuals (23 males, 17 females, and 9 nonadults). In addition, we explored possible intraindividual lifetime changes by comparing collagen *δ* ^13^C from bone and dentine of 26 individuals. We assessed nonlocality based on individual deviation of isotopic values from the population mean plus three times the median absolute deviation from the median (±3MAD). We then checked for isotopic differences between sexes and type of funerary treatment using Mann–Whitney tests.

**Results:**

One individual shows isotopic values consistent with a nonlocal origin. Five more individuals may have originated from a different locality. No statistical differences separate sexes and types of funerary treatment.

**Discussion:**

Results suggest a local origin of most of the individuals of SV with the few exceptions pointing especially to an Alpine origin. The low frequency of nonlocals at SV suggest a reduced mobility in this population, or the preeminence of short distance movements undetected by our analyses.

## Graphical abstract

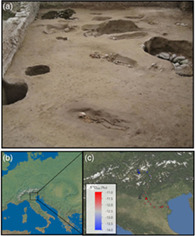

## 1 INTRODUCTION

The reconstruction of human mobility and migration in the past is a traditional research topic in anthropology and archaeology. This is especially due to the biological, social, and cultural relevance of these processes, and the involved demographic, economic, social, cultural and political factors (Adey, 2010; Leary, 2014).

Nowadays a standard analytical tool in paleomobility studies is the application of isotopic analyses. The application of these techniques has provided various insights about human movements for a large number of archeological contexts (e.g., Bentley, 2013; Chenery et al., 2010; Fernández‐Crespo et al., 2020; Francisci et al., 2020; Frei et al. 2017; Lightfoot et al., 2014; Lösch et al., 2014; Milella et al., 2019; Paladin et al., 2020; Redfern et al., 2016; Siebke et al., 2020; Stark et al., 2020 etc.). Especially during the last decade, a series of isotopic studies have explored mobility and migration in continental Europe during the Late Iron Age (ca. 4th–1st c. BCE) (Grupe et al., 2020; Hauschild et al., 2013; Knipper et al., 2014, 2018; Moghaddam et al., 2016; Moghaddam et al., 2018; Müller‐Scheeßel et al., 2020; Oelze et al., 2012; Scheeres et al., 2013, 2014; Sorrentino et al., 2018). This body of research has provided important information about human mobility, especially for Central Europe. Nonetheless, our understanding of human movements and of their sociocultural correlates during this period is still fragmentary, especially for the regions to the south of the Alpine range.

The Late Iron Age (culturally denominated “La Tène”) of Continental Europe is a period characterized by intense demic and cultural exchanges between the regions north to the Alps (Transalpine areas) and those to the south of the Alpine range, like Northeastern Italy (Cisalpine area) (Kruta, 2009; Vitali, 2004, 2011). These processes are consistent with the economic relevance of the alpine areas since Prehistory (Gilck & Poschlod, 2021; Hafner & Schwörer, 2018; Putzer et al., 2016), and with its role as a gateway for the transit of objects, people and ideas. For the Iron Age, this is demonstrated by the southward diffusion of material culture (e.g., La Tène swords and brooches) originating in the transalpine regions (Kruta, 2009; Vitali, 2001). Modern archeological theories are increasingly critical about traditional hypotheses about one‐way mass migrations at the basis of the observed archeological patterns, rather privileging more nuanced interpretation centered on individual or small group moves and gradual processes (Anctil, 2021 and references therein; Isayev, 2017). In any case, the available data would agree with ancient Greek sources (Appianus, Dionysius of Halicarnassus) in placing the earliest important presence of “Celtic/Latenian” populations in the Italian Peninsula around the 4th century BCE (Grassi, 2009; Kruta, 1977, 1988).

Little is known about the lifestyle, cultural affiliation, and ethnic origin of the “Celtic” groups distributed in the Italian peninsula between the 4th and 1st centuries BCE. Moreover, historical, archeological, and anthropological data are available especially for some of these groups. These include the Boii, the Cenomani, and the Insubri in the North of the Italian peninsula, and the Senoni in the central regions (Grassi, 2009). These populations were characterized not only by their distinct regional distribution and cultural traditions, but also by heterogeneous relationships (conflicts, alliances, peaceful coexistence) with indigenous Italic, Etruscan, and Roman groups (Gambacurta, 2013; Grassi, 2009). Previous anthropological studies on “Celtic” groups in the Italian Peninsula have especially focused on Boii and Cenomani. The former, distributed in nowadays Emilia Romagna region have been the subject of paleopathological analyses aimed at reconstructing patterns of health and well‐being (Brasili, 1992; Brasili et al., 2000) and of paleomobility isotopic studies (Scheeres et al., 2013; Sorrentino et al., 2018). The study by Scheeres et al. (2013) focused on strontium and oxygen isotopic ratios, whereas the analysis of Sorrentino et al. (2018) included a comparison of isotopic (strontium), phenetic (dental non‐metrics traits), and archeological variables (funerary variability). Both studies revealed a high proportion of male nonlocal individuals in the analyzed samples, and to several individuals who moved already during childhood. These results offer some insights into the possible socioeconomic features of these populations. Particularly, they seem to suggest (a) a dynamic situation featuring a relatively high frequency of movements, and (b) differences in the latter between sexes. These conclusions confirm expectations based on geographic, historical, and archeological data. The latter point throughout the Iron Age, to a mosaic of contacts and cultural exchanges not only between “Celtic” and local italic populations but also between Celtic groups distributed on the two sides of the Alpine range (Butti Ronchetti, 2014; Marzatico, 2014; Ramsl, 2014; Vitali, 1996, 2001). Various causes were likely at the basis of these movements. Classical sources, for example, stress the role played by economic and commercial factors, the attraction on transalpine cultures exercised by Mediterranean products such as grapes, figs, wine, and oil, and the need to appropriate new lands for cultivation (Mansuelli, 1978; Vitali, 2011).

Among the economic drivers of mobility, one needs also to include mercenary service, an activity traditionally attributed to these groups (Hauschild, 2013; Vitali, 2011). Especially during the 3rd century BCE, individuals moved across the Alpine range in the service of third parties (Polybius II, 19, 1–4 and II, 34, 21 in Vitali, 2011).

When we shift our focus to the Northeast of the Italian Peninsula, bioarchaeological data on human movements during the late Iron Age are completely lacking. The work by Laffranchi et al. (2016) and Laffranchi and colleagues (Laffranchi et al., 2015, 2016, 2018, 2019; Laffranchi, Charisi, et al., 2020) has contributed to the reconstruction of the diet, exposure to biomechanical and nonspecific stressors, gender roles and social differentiation among the Cenomani (Verona area). From the same group, however, no data are yet available about the presence and frequency of nonlocal individuals, and about the associated mobility and migration processes.

### 1.1 Insights into past human movements from intraskeletal variability of oxygen and carbon stable isotope ratios

Together with those of strontium (^87^Sr/^86^Sr), isotopic ratios of oxygen (*δ* ^18^O) are at the basis of most paleomobility research. By means of ingestion of drinking water and water contained in food, dental and skeletal phosphate acquire during their formation the *δ* ^18^O signature of the surrounding environment (D'Angela & Longinelli, 1990, 1993; Delgado Huertas et al., 1995; Longinelli, 1984). The isotopic composition of meteoric precipitation displays a strong geographical trend, being influenced by a set of variables including latitude, distance from the coast, altitude, temperature, and humidity (Lightfoot & O'Connell, 2016; Rozanski et al., 1993). Consequently, estimates of water *δ* ^18^O based on phosphate isotopic composition can be used to reconstruct past human movements and/or palaeohydrological changes (D'Angela & Longinelli, 1990, 1993; Delgado Huertas et al., 1997; Lightfoot et al., 2014; Lightfoot & O'Connell, 2016; Reynard & Hedges, 2008). Specifically, a deviation in the isotopic signals of dental or skeletal tissues from those of the burial location may provide hints about the possible nonlocal origin of an individual. This interpretation, however, needs to take into account the possible effect of various factors on the *δ* ^18^O values of water sources. These include, for example, evaporation of surface water and the access of humans and/or animals to rivers, groundwater, local rainwater pools preserving the isotopic signal of a different location (e.g., rivers originating at higher altitudes) (Gat, 1971; Lightfoot & O'Connell, 2016; Pederzani & Britton, 2019).

Oxygen stable isotopes in archaeology are commonly measured in tooth enamel or bone bioapatite (carbonate and phosphate). Compared with carbonate, phosphate offers the advantage of being less prone to taphonomic/diagenetic alterations (Britton et al., 2015; Delgado Huertas et al., 1997; Francisci et al., 2020; Kohn & Cerling, 2002; Luz & Kolodny, 1989). On the other hand, when compared with that from dental enamel, the isotopic composition of bone phosphate is the weighted average of the last 10–25 years of life (Longinelli, 1984; Manolagas, 2000). This plays an obvious role in the type of information that the analysis of these tissues can provide in paleomobility research. Although less frequently applied, isotopic analyses of oxygen in bone phosphate have yielded interesting results in bioarchaeology. Especially for precontact Meso and South American populations, the application of this method has provided important information about the degree of residential mobility in these contexts (Moreiras Reynaga et al., 2021; Toyne et al., 2014; Webb et al., 2013; White et al., 2000, 2004).

Stable isotopes ratios of carbon (*δ* ^13^C), although usually applied in paleodietary research, can also provide insights into paleogeography (Eerkens et al., 2014; Hakenbeck et al., 2010). Different types of plants (C_3_ vs. C_4_) may indeed present a variable spatial distribution because of different climatic and environmental conditions and/or different cultivation practices. C_3_ plants (e.g., wheat, barley, and the majority of plants and fruits) are typical of cool and wet environments, whereas C_4_ plants (e.g., millet, sorghum, maize, etc.) are well adapted to warm climates, and rare in Europe, with millet being the only known domesticated one during Prehistory. The relative abundance of C_3_ versus C_4_ plants is therefore highly correlated with, and therefore informative about, climatic factors (temperature, precipitation) as well as cultivation strategies (Ehleringer et al., 1997; Laffranchi et al., 2016; Lösch et al., 2006; Teeri & Stowe, 1976). *δ* ^13^C ratios can also provide information about the consumption of freshwater/marine vs. terrestrial resources (DeNiro & Epstein, 1978; Schoeninger & DeNiro, 1984; Smith & Epstein, 1971). Individual *δ* ^13^C values deviating from those of the rest of the population may point to the consumption of different foodstuffs, either due to cultural or social reasons (e.g., intrapopulation access to C_4_ vs. C_3_ plant products based on status, gender, or age) or because of a different geographic origin. The paleoecological information content of *δ* ^13^C, suggested by a large literature (among others DeNiro & Epstein, 1978; Van der Merwe, 1982; Schoeninger & Moore, 1992) suggests its potential use as a complementary tool in paleomobility research.

When trying to reconstruct past diet and mobility, the interpretation of isotopic data can be informed by intraskeletal differences in collagen turnover rate. Ribs show in general a faster turnover rate than other commonly sampled bones like femur and tibia (Fahy et al., 2017; Hill & Orth, 1998; Parfitt, 2002). The turnover of dentine collagen is, conversely, extremely slow, and its isotopic signal reflects the diet during the time of dentine formation (Beaumont et al., 2013). Accordingly, the analysis of collagen from dentine and from different skeletal elements with different remodeling rates can provide information about intravitam patterns in access to specific foods and/or residential patterns.

Such an approach has already provided useful insights from a large series of archeological contexts (Cheung et al., 2017; Cox & Sealy, 1997; Frei et al., 2015; Hedges et al., 2007; Lamb et al., 2014; Müller et al., 2003; Pollard et al., 2012; Schroeder et al., 2009; Sealy et al., 1995; Toyne et al., 2017, 2021; Tsutaya et al., 2016; Varalli et al., 2021).

### 1.2 Archeological and anthropological background

The necropolis of Seminario Vescovile (henceforth SV) was discovered and excavated between 2005 and 2010 in Verona (NE Italy). Preliminary typological analysis of grave goods related this context with the pre‐Roman/Celtic culture of the Cenomani. The Cenomani are scarcely documented in the archeological literature, and almost no information is available regarding their geographic origin. The only historical data come from Livius (Livius, Ab Urbe Condita, V, 35.1), who refers to their settling in the area between the modern cities of Brescia and Verona during the 5th–4th centuries (Kruta & Manfredi, 1999; Malnati et al., 2004). Throughout the 2nd and 1st centuries BCE this population was progressively integrated into the Roman cultural and political sphere (Cavalieri Manasse, 2004; Grassi, 2009; Malnati et al., 2004). Radiocarbon datings placed the use of SV between the 3rd and 1st century BCE (Laffranchi, 2016; Laffranchi et al., 2015, 2016, 2019).

**FIGURE 1:**
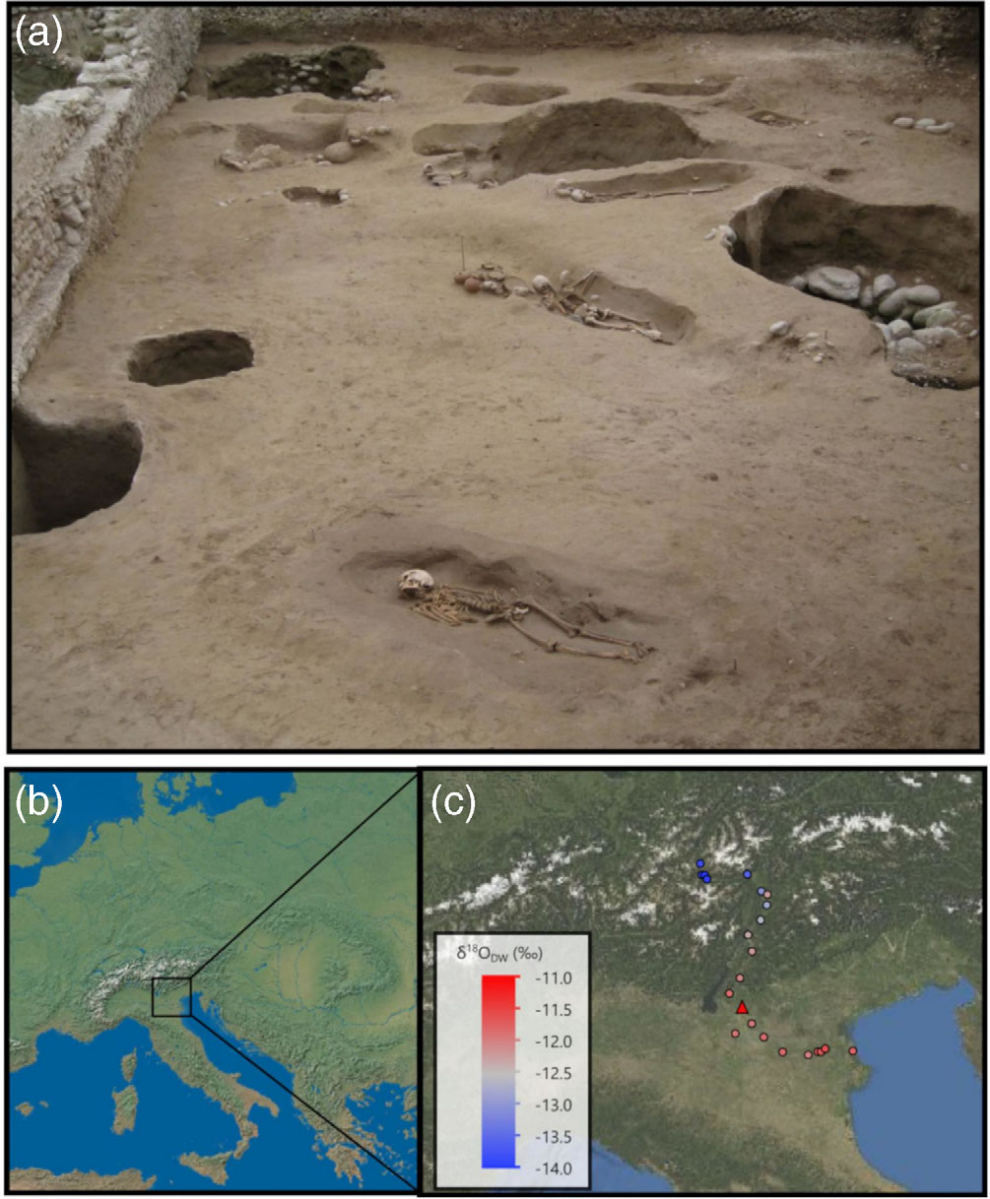
(a) View of SV upon excavation (photo by S. Thompson, by courtesy of SABAP‐VR Soprintendenza archeologia, belle arti e paesaggio per le province di Verona, Rovigo e Vicenza); (b) estimated mean *δ* ^18^O_dw_ value for SV (triangle) and mean *δ* ^18^O water values from various sampling stations along the Adige river (data from Natali et al., 2016)

With a minimum number of 174 inhumated individuals, SV (Figure 1a and b) stands out as one of the largest and better‐preserved “Celtic” necropolises in the Italian peninsula. Burials include mostly supine inhumations, with a small number of individuals either prone or on their side (see Table S1). In some cases, burials are provided with “funerary structures” represented by stones marking the edge of the pit and/or covering the burial surface (cf. Laffranchi et al., 2019 for further details).

Grave goods variability (e.g., pottery, pins, coins, rings, and few knives, but no weaponry) suggests a rather homogenous society, with no significant differences between sexes in the type and amount of grave goods, and an apparent absence of vertical social differences in burial treatment (see also Laffranchi, 2016; Laffranchi et al., 2019; Laffranchi, Charisi, et al., 2020).

An intriguing exception is US 2807, the burial of a middle adult male standing out from the rest of the population for its unique archeological and anthropological features (Laffranchi et al., 2015). These include (1) his provision with a grave good item (a probable wooden tub or barrel) that is unique at SV; (2) the oldest ^14^C datation for now at SV (2201–2137 cal. BP), and (3) the co‐presence of several congenital anomalies (foot polydactyly, bipartite medial cuneiform, dental agenesis—Laffranchi et al., 2015).

A recent series of anthropological and isotopic works have revealed further details about the lifestyle and diet of this population. Patterns of entheseal changes and long bone shape and robusticity suggest differences between the sexes in the performance of daily activities and general exposure to biomechanical stress (Laffranchi, Charisi, et al., 2020). Further biocultural data have been provided by analyses of linear enamel hypoplasia, stable isotopes ratios of carbon and nitrogen, and funerary variability (Laffranchi et al., 2016, 2018, 2019). Overall, the picture emerging from these studies is that of a population featuring a weak social differentiation and whose members were equally exposed to developmental stressors during growth (Laffranchi et al., 2019). Diet at SV was characterized by a prolonged breastfeeding period (Laffranchi et al., 2018), and almost exclusive reliance on cultivated crops (C_4_ plants, possibly broomcorn and foxtail millet). The diet of males featured a higher intake of animal protein, whereas that of females was characterized by a higher intake of cereals and vegetal proteins (Laffranchi et al., 2016).

Although no previous study investigated human mobility at SV, various factors (geographical, orographic, and archeological) make such an analysis worthwhile. First, the region stands at the crossroad between the Alpine range and the Southern Po Plain and is nowadays only 90 Km away from the Italian Adriatic coast. It features a relatively mild climate, fertile soils, and is crossed by an important waterway (the Adige River) which stands as a natural connection between the Cisalpine and Transalpine areas. The Adige River originates near the Reschen Pass (South Tyrol), has a course of more than 400 Km for finally flowing into the Adriatic Sea. The economic, military, and cultural relevance of the area surrounding SV is further demonstrated by the archeological traces of its occupation before, during, and after Roman times (Cavalieri Manasse, 2004; Laffranchi, 2016; Malnati et al., 2004). The archeological traces of coexistence and cultural contact between the Cenomani and the local Venetic populations, with transalpine groups, and, toward the last centuries BCE, with the Roman cultural sphere, contribute to this dynamic picture (Gambacurta & Ruta Serafini, 2019; Marinetti, 1992; Prosdocimi, 1991; Vitali, 2001). All these factors make the Northeast of the Italian peninsula in general, and the region surrounding SV in particular, of great interest from a paleomobility perspective. This was further suggested by previous analyses of *δ* ^13^C at SV. In the context of rather homogenous population values (mean of −15.3‰ ± 2.2‰ V‐PDB), some individuals indeed showed markedly depleted ratios (between −20 and −19‰ V‐PDB), suggestive of their possible nonlocal origin (Laffranchi, 2016; Laffranchi et al., 2016). This interpretation was based on various eco‐geographical and cultural factors: (a) the relative proximity of SV to subalpine and alpine regions, (b) the historically documented transalpine origin of Cenomani, and (c) the archeological traces documenting contacts between cisalpine and transalpine cultural spheres throughout the 4th‐1st centuries. These considerations raise the likelihood that some individuals relocated to SV from more Northern and higher altitude areas. The latter present environmental and climatic characteristics that make them less suitable for the cultivation of millet (Ehleringer et al., 1997; Laffranchi et al., 2016; Paladin et al., 2020). One would therefore expect these nonlocals to show a comparatively depleted carbon isotopic signature, consistent with larger consumption of C_3_ plants. Admittedly, and lacking additional data, this has however remained an exploratory hypothesis deserving further study. Based on these premises, the aim of this work is to explore the presence of nonlocal individuals at SV by means of an analysis of stable isotopes ratio of oxygen from bone phosphate and carbon from bone and dentine collagen. Specifically, our study tries to address three main research questions:

1. What is the frequency of potential nonlocals at SV, and what does it tell about the type of human movements taking place in this population?
2. Is there any pattern among assumed nonlocals based on sex and/or age?
3. Is there an association between the isotopic signature of nonlocality and the type of funerary treatment?

## 2 MATERIAL AND METHODS

Table 1 shows the distribution of the sample by age and sex. We analyzed 49 individuals representing both sexes and different age classes. The detailed demographic distribution of SV has been previously published by Laffranchi et al. (2016), Laffranchi et al. (2019), and Laffranchi, Charisi, et al. (2020). Adult age‐at‐death was estimated based on the morphological changes of the pubic symphysis, of the auricular surface of the ilium, and of the sternal end of the 4th left rib or others in case of absence (Brooks & Suchey, 1990; Buckberry & Chamberlain, 2002; İşcan et al., 1984, 1985). We estimated nonadults' age‐at‐death based on the development and eruption of deciduous and permanent teeth, using diaphyseal measurements, and based on the degrees of epiphyseal fusion (Schaefer et al., 2009; Scheuer & Black, 2000; Ubelaker, 1989). Sex was determined based on the morphology of pubic symphysis, coxal bones, and cranial and mandibular dimorphic traits, following standard anthropological methods collected in Buikstra and Ubelaker (1994).

**TABLE 1:** Age and sex distribution of the analyzed individuals

	Females		Males		Sex: NA		Total	
	*n*	%	*n*	%	*n*	%	*n*	%
YA	9	52.9	11	47.8	0	0	20	40.8
MA	8	47.1	12	52.2	0	0	20	40.8
NaI	0	0	0	0	2	22.2	2	4.1
NaII	0	0	0	0	3	33.3	3	6.1
NaIII	0	0	0	0	1	11.1	1	2.0
NaIV	0	0	0	0	3	33.3	3	6.1
Total	17	100	23	100	9	100	49	100

*Note*: NaI: ca. 37–42 weeks old; NaII: (0–1 year old); NaIII: (>1 to 5 years old); NaIV: (>5 to 14 years old); YA: young adults (19–34 years old); MA: middle adults (35–50 years old). Sex: NA: individuals for which it was not possible to determine sex.

Following our previous work (see Laffranchi et al., 2019), we grouped nonadults in NaI (ca. 37–42‐weeks‐old), NaII (0–1‐year‐old), NaIII (>1–5‐years‐old), NaIV (>5–14‐years‐old). Adults were subdivided in Young Adults (19–34 years old) and Middle Adults (MA: 35–50 years old). Five individuals were possibly aged over 50 years old, but were included in the MA age class in order to avoid unbalances in sample sizes and associated issues during statistical testing.

### 2.1 Stable isotopes analysis (δ^13^C)

Stable carbon and nitrogen isotope ratios from rib bone collagen from SV are published and discussed elsewhere (see Laffranchi et al., 2016, 2018, 2019). We refer the reader to these works for a detailed description of the applied standards and analytical protocols. This study is based on the analysis of 49 individuals for whom both carbon and oxygen data are available. Here we add data for a subsample of 26 adult individuals from whom we processed dentine collagen. The latter was obtained from the dental root (for the major part second premolars and second/third molars), sectioned transversely straight below the crown. In order to minimize the breastfeeding signal, we selected mostly premolars and second/third molars since these teeth start to mineralize during the first years of life and continue to grow until adolescence and early twenties (for M3) (Scheid, 2007; Schroeder et al., 2009). Collagen extraction followed the protocol of Bocherens et al. (1991, 1997) and the quality criteria suggested by Ambrose (1990, 1993) Van Klinken (1999), and DeNiro (1985). We included samples with >1% collagen yield, molar C:N ratio in the range of 2.9–3.6 (DeNiro, 1985), %C between 30% and 47% and %N between 11% and 17.3% (Ambrose, 1990; Van Klinken, 1999). Our aim in analyzing both bone‐ and dentine collagen was to explore possible isotopic differences between tissues characterized by different turnover rates and therefore informative about different times during the lifetime of an individual (Sealy et al., 1995; Toyne et al., 2017, 2021). If present, these differences may inform about dietary changes throughout an individual's lifetime. The latter, besides their possible link to cultural practices or personal preferences, may also result from individual moves between areas featuring different eco‐geographical conditions (see also Eerkens et al., 2014; Hakenbeck et al., 2010; Schroeder et al., 2009). The unique features of inhumation US2807 (see Introduction) suggested to analyzed two different teeth (URM2 and LLP4), in order to explore two different phases of the individual's childhood. The dentine closest to the crown margin of M2 forms around 3 years, after initial enamel calcification, followed by formation toward the pulp cavity between ~4.5 and 7.5 years. The enamel calcification of lower P4 is completed around the age of 7 years; the root (and the dentine) continues to form until the age of 14 years (Hillson, 1996; Lamb et al., 2014). The dentine isotopic values of inhumation US2807, namely URM2 and LLP4 should therefore provide information for the age periods roughly between 3 and 7.5 years and subsequently between 7 and 14 years.

Although nitrogen isotope ratios are available for all individuals, in this study we decided to focus only on δ^13^C. The potential bias played on δ^15^N by the breastfeeding signal would indeed be too elevated when comparing dentine and bone collagen isotopic values. This issue is also confirmed when screening the isotopic ratios from these two tissues (see Table S1). Our choice to focus on *δ* ^13^C is also based on the use of carbon as an accessory tracer of mobility by other studies.

Henceforth, we will refer to *δ* ^13^C from bone and dentine collagen as *δ* ^13^C_bo_ and *δ* ^13^C_de_ respectively. Analysis of nitrogen and carbon ratios was carried out by means of a Carlo Elba NC1500 (Milan, Italy) elemental analyzer on line with a Delta Plus XP (ThermoQuest, Bremen, Germany) mass spectrometer (EA‐IRMS). Commercial CO_2_ and N_2_ were used as the internal standards for the carbon and nitrogen isotopic analyses (see Laffranchi et al., 2016, 2019 for more details).

### 2.2 Oxygen (δ^18^O) analysis

The protocol to analyze oxygen stable isotopes in bone phosphates follows a complex chemical treatment to purify the samples and to precipitate them as silver phosphate (Ag_3_PO_4_) (Crowson et al., 1991; Longinelli, 1965; Tudge, 1960). The preparation of the samples and the MS‐ measurements were carried out at the Stable Isotope Laboratory of the *Instituto Andaluz de Ciencias de la Tierra* (CSIC‐UGR, Granada). Bone phosphate is known to be particularly resistant to diagenetic processes. This is for example demonstrated by analyses of oxygen isotopic ratios from bone phosphate of samples dating 13.000–30.000 years old (Delgado Huertas et al., 1997).

Nonetheless, in this study, we checked for different criteria in order to authenticate our phosphate isotopic values. First, we selected well‐preserved bone samples from ribs showing no visual signs of dissolution or recrystallization. The good preservation of the samples, already preliminarily observed macroscopically, and attributable to the fine sand in which the skeletons were found (Laffranchi, 2016), is also suggested by their collagen isotopic data (C and N), which fit the quality criteria suggested by Ambrose (1990, 1993) and DeNiro (1985) (see Table S2).

In order to check for recrystallization processes, we then analyzed five samples by means of a PANalytical X' Pert Pro diffractometer for powder samples (XR Diffraction Unit of the *Instituto Andaluz de Ciencias de la Tierra* ‐CSIC‐UGR, Granada). We selected for this analysis the two samples showing respectively the more positive and more negative isotopic ratios and three additional ones showing values in between these extremes.

For each sample we selected 200–300 mg of bone powder, we removed the organic matter by treating the samples with undiluted hydrogen peroxide (H_2_O_2_), and left them for a week at room temperature by stirring them several times a day with a Vortex ZX Classic mixer. This process was repeated several times, renewing the H_2_O_2_ for a few weeks until finally the samples were placed in an oven at a low temperature to completely evaporate the H_2_O_2_. Successively, 2 ml of 2 M hydrofluoric acid (HF) was added, and after 24 h, the calcium fluoride (CaF_2_) was formed, leaving the PO_4_ in solution. After the centrifugation (to separate the solid), we added to the resulting solution (without solid) 2.2 ml of 2 M potassium hydroxide (KOH). This solution was then treated with a buffer of Silver Nitrate (AgNO_3_) + Ammonium Nitrate (NH₄NO₃) + Ammonium Hydroxide (NH₄OH) and put into a thermostatic bath (Julabo sw23) at a constant temperature of 50° (increasable up to 70°). During this process, NH_3_ is released and the phosphates crystallize (Ag_3_PO_4_), afterward the samples (pH less than 7.5 but not less than 6.8) were filtered and dried. An internal standard of bone phosphate, magmatic apatite, and phosphorite with known isotopic composition was precipitated (following the same process described above) to verify that no isotopic fractionation has occurred during the precipitation process.

Samples (Ag_3_PO_4_) mixed with graphite were placed in silver capsules that fall onto a ceramic column containing a glassy carbon tube at 1450°C to produce CO (Sharp et al., 2001); this system is coupled on‐line via a ConFlo IV interface to a Delta V Plus isotope ratio mass spectrometer (Thermo‐Finnigan, Bremen). The CO was separated by chromatography using a helium carrier gas stream. Commercial CO and five different internal and international standards were used for the oxygen isotopic analyses. To avoid memory effects, each sample was analyzed six times. We discarded the first three analyses and calculated the average of the last three. For oxygen 12 internal standards (organic and inorganic material, including Ag_3_PO_4_) ranging between −27.93‰ and +71.4‰ (V‐SMOW), contrasted with the IAEA international references, IAEA‐NO‐3, IAEA‐C3, NBS‐127, USGS35 and USGS34 are commonly used. For this study three internal standards of +30.2‰, +8.7, and −27.93‰ (V‐SMOW) have been used. After correction of the mass spectrometer daily drift, the calculated precision from standards systematically interspersed in analytical batches was better than ±0.2‰ for phosphate oxygen. The standard for reporting oxygen is V‐SMOW.

To approach the geographical localization of the burials, phosphate *δ* ^18^O_bo_ values (V‐SMOW) were subsequently converted to the oxygen isotopic composition of drinking water, also referred to as meteoric water (*δ* ^18^O_dw_ vs. V‐SMOW). For this, we used the equation provided by Daux et al. (2008): *δ* ^18^O_dw_ = 1.54 (± 0.09) × *δ* ^18^O_p_ − 33.72 (± 1.51). These values were then compared with published *δ* ^18^O water data from the river Adige (D'Angela & Longinelli, 1993; Müller et al., 2003; Natali et al., 2016; Toncala et al., 2017), from precipitation across Italy (Bowen, 2021; Giustini et al., 2016; IAEA/WMO, 2015; Longinelli & Selmo, 2003), and with isotopic ratios obtained from the sampling of local waters. Specifically, we analyzed *δ* ^18^O (and *δ*D) from ten water samples representing two different altitudinal points along the course of the Adige river, three tributaries of the Adige near Verona, as well as local water sources, well‐ and tap waters from the surroundings of SV (Table S3). The isotopic analyses of the water samples were carried out by injecting 1.8 microliters into a Picarro L‐2140i. The replications of internal standards (contrasted with IAEA international standards) indicate errors of less than 0.1‰ and 0.5‰ for *δ* ^18^O and *δ*D, respectively.

### 2.3 Statistical protocol

We first screened our data for the presence of isotopic outliers in *δ* ^18^O_bo_, *δ* ^13^C_bo_, and *δ* ^13^C_de_, by using as criterion the burial communities median added and subtracted three times the median absolute deviation from the median (henceforth 3MAD) (Lightfoot & O'Connell, 2016; Milella et al., 2019). We performed the same procedure focusing on the difference between *δ* ^13^C_bo_ and *δ* ^13^C_de_ (henceforth ∆^13^C_bo−de)_. We also tested for differences between *δ* ^13^C from bone and dentine collagen comparing the two datasets with a Mann–Whitney *U* test.

Previous analyses highlighted a significant difference in *δ* ^13^C_bo_ between sexes, a result pointing to a higher contribution of cereals and vegetal proteins to the diet of females (see Laffranchi et al., 2016). To minimize the possible bias of dietary differences on our data, we, therefore, decided to compare sexes for *δ* ^18^O_bo_ and ∆^13^C_bo−de_ using Mann–Whitney *U* tests.

We then grouped all individuals according to the presence or absence of three basic funerary variables: the presence or absence of grave goods, the position of the skeleton (supine or not), and the presence of funerary structures (see introduction).

We then compared the resulting groups for *δ* ^18^O_bo_ and ∆^13^C_bo−de_ through Mann–Whitney *U* tests. We performed all analyses and plots in JMP, setting alpha = 0.05.

## 3 RESULTS

**FIGURE 2:**
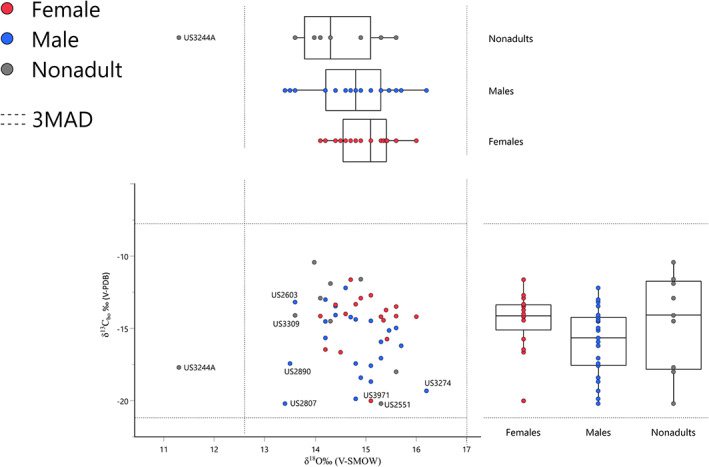
Plot of the analyzed individuals along *δ* ^18^O_bo_ and *δ* ^13^C_bo_, and distribution of adults and nonadults for each isotopic ratio. Dotted lines indicate the upper and lower 3MAD isotopic ranges. US numbers indicate the individuals discussed in the text

**FIGURE 3:**
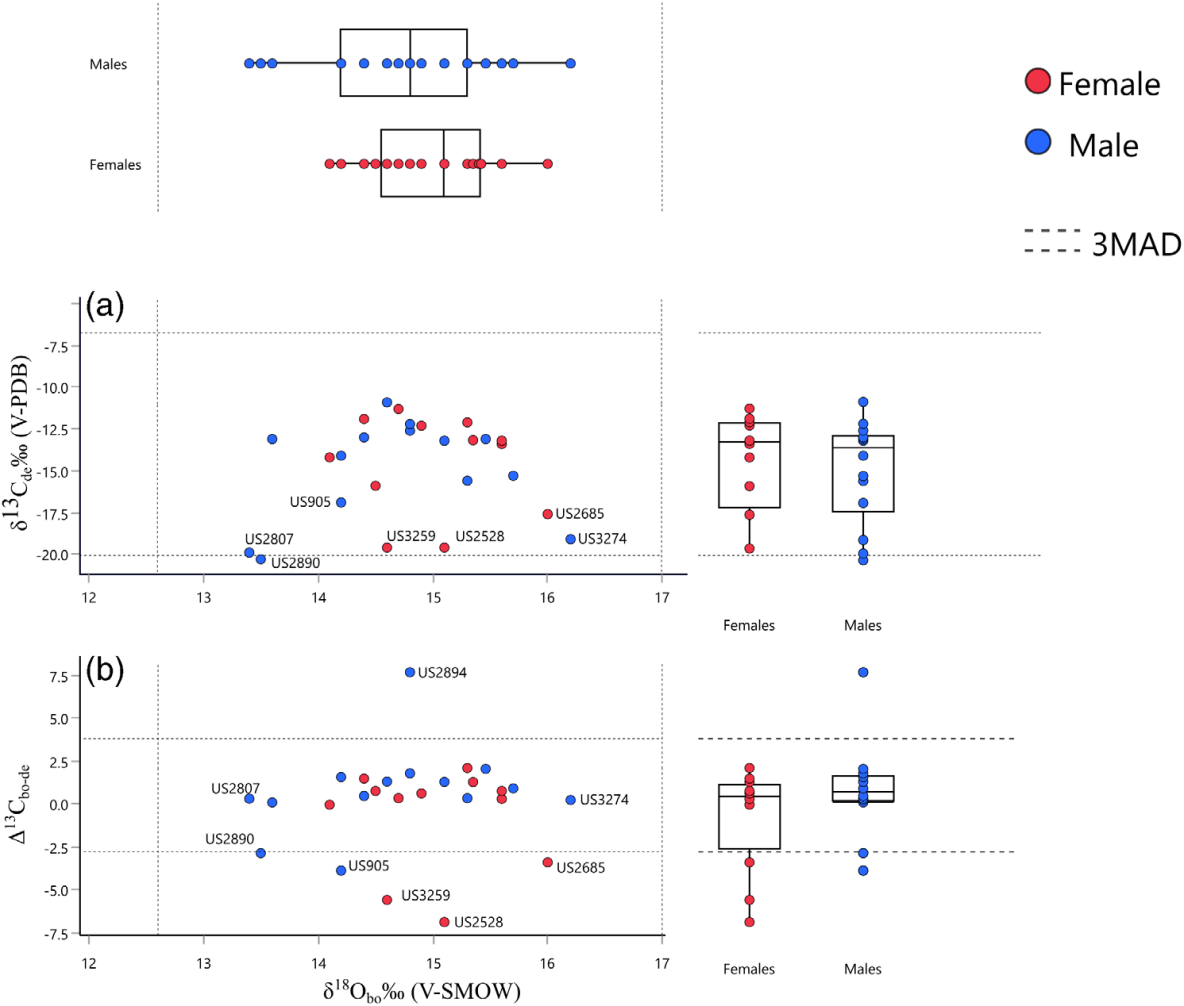
Plot of *δ* ^18^O_bo_ versus *δ* ^13^C_de_ (a), and *Δ* ^13^C_bo−de_ (difference between *δ* ^13^C values from bone and dentine collagen) (b). Boxplots visualize the distribution of each isotopic variable according to sex. Dotted lines indicate the upper and lower 3MAD isotopic ranges. US numbers indicate the individuals discussed in the text

Table S1 presents all demographic, isotopic, and funerary data. Figures 2 and 3 plot the ranges of *δ* ^18^O_bo_, *δ* ^13^C_bo_, *δ* ^13^C_de_, and ∆^13^C_bo−de._

### 3.1 Assessment of sample preservation

All carbon and nitrogen isotopic values fit the quality criteria and these are presented in Table S2, as recommended by Szpak et al. (2017). Individuals US3274 and US3244A show the more positive and more negative *δ* ^18^O_bo_ values, respectively. The X‐ray diffraction patterns of these samples and the additional three (US 2807, US 3309, and US 3243) selected from those falling inside this range do not show the presence of recrystallization processes (see Figure S1).

### 3.2 Oxygen isotopic ratios

*δ* ^18^O_bo_ ranges from 16.2‰ to 11.3 ‰ (mean: 14.7 ± 0.8‰ V‐SMOW). Only one individual (US3244A, NaII) with a markedly depleted isotopic signature (11.0‰ V‐SMOW), falls outside the ±3MAD range, (Figure 2). Even though not statistical outliers, four additional individuals (US2603, 2807, 2890, and 3309) present particularly depleted values. These include three males (one young adult and two middle adults) and one infant of 2–3 months. Another male (US3274: middle adult) presents the most enriched *δ* ^18^O_bo_ value (16.0 ‰) (Figure 2). When converted to *δ* ^18^O_dw_ these values correspond to a sample average of −11.03 ± 1.3‰. Converted values for individuals US3244A, 2603, 2807, 2890, 3309, and 3274 are −16.3‰, −12.8‰, −13.1‰, −12.9‰, −12.8‰, and −8.8‰ V‐SMOW, respectively.

Oxygen isotopic ratios from our water samples range from −7.7‰ (Avesa stream, Verona) to −12‰ (Adige River, upper course Adige River) (Figure S2 and Table S3).

### 3.3 Carbon isotopic ratios

*δ* ^13^C_bo_ and *δ* ^13^C_de_ range respectively from −21.2‰ to −7.7‰ (mean: −15.15 ± 2.8‰ V‐PDB), and from −20.3‰ to −6.7‰ (mean: −14.7 ± 3.4‰ V‐PDB). We do not find outliers for *δ* ^13^C_bo_ although we point out four individuals, with particularly depleted *δ* ^13^C_bo_ values. These are US3971 (young adult female), US2551 (nonadult, NaIV), and US2807 (middle adult male) (Figure 2). The only outlier for *δ* ^13^C_de_ with a particularly depleted isotopic value (−20.3‰ V‐PDB) is US2890 (young adult male) (Figure 3a).

Males and females show similar ranges for *δ* ^13^C_de_, whereas for *δ* ^13^C_bo_ females show less dispersed values, with one individual (US3971, young adult female) falling well outside the isotopic range for this sex (−20.0‰ V‐PDB).

**FIGURE 4:**
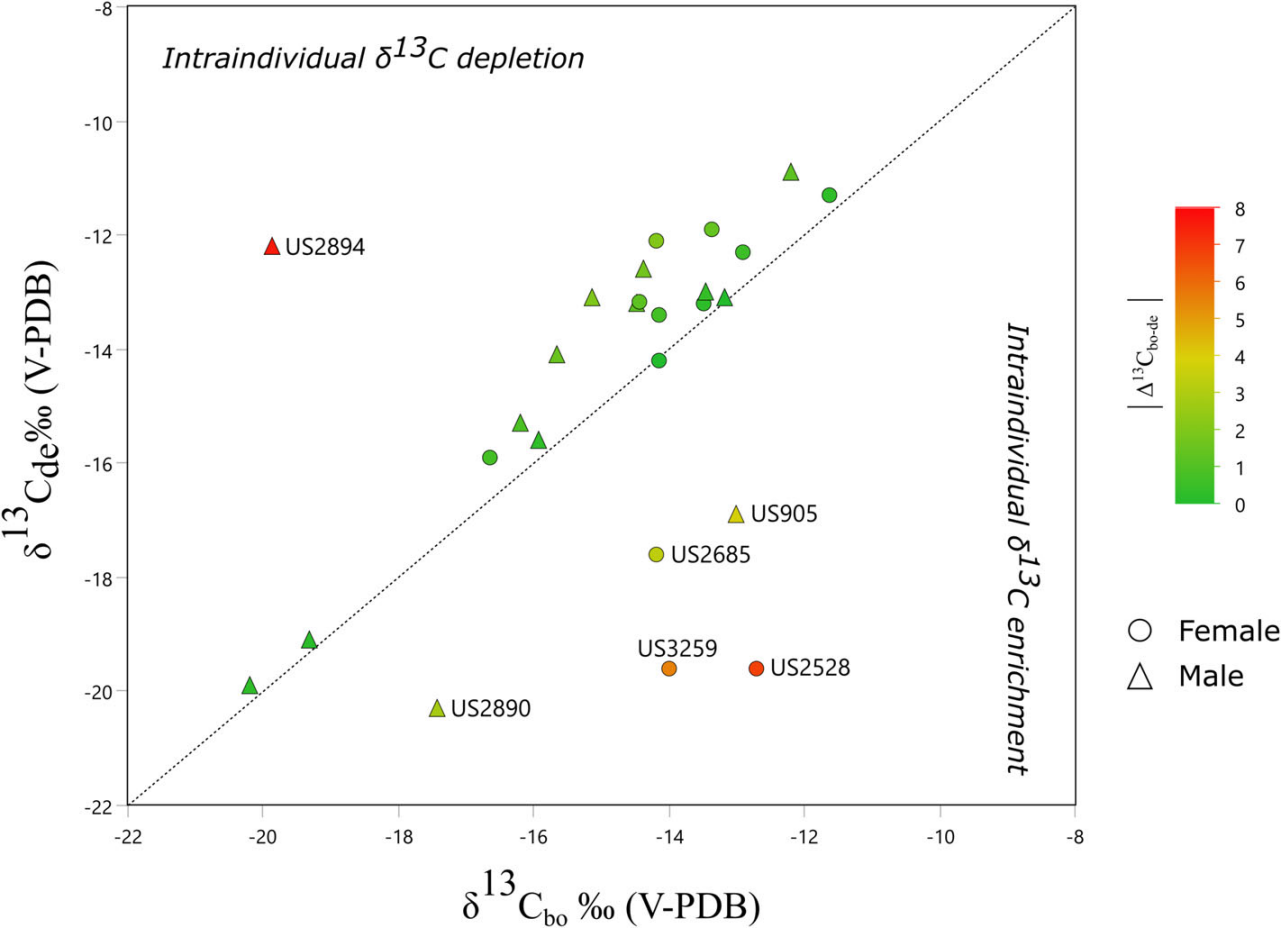
Plot of *δ* ^13^C_bo_ versus *δ* ^13^C_de_. Male and females are indicated by triangles and circles, respectively. Individual deviations from a theoretical perfect positive correlation between *δ* ^13^C_bo_ and *δ* ^13^C_de_ are further highlighted by means of a color code expressing the absolute difference between variables

Individual ∆^13^C_bo−de_ values present an interesting variability (Figure 3b and 4). First, most of the sample falls in a rather narrow range (mean: 0.1 ± 2.8), pointing to, on average, a minimal isotopic shift between bone and dentine. This is further demonstrated by the lack of statistically significant differences between the two types of samples for *δ* ^13^C (Table S4). Three individuals, however, stand out for their large ∆^13^C_bo−de_ values. Two females (US3259 and US2528, both young adults) show *δ* ^13^C_bo_ values that are enriched compared with their *δ* ^13^C_de_ values (Figures 3b and 4). The opposite situation (*δ* ^13^C_bo_ depleted compared with *δ* ^13^C_de)_ characterizes the middle adult male US2894. Smaller values of ∆^13^C_bo−de_ are also shown by US2890 and US905 (young adult males) and US2685 (middle adult female) (Figures 3b and 4).

Formal statistical tests show no significant difference between sexes or funerary features for ∆^13^C_bo−de_ and *δ* ^18^O_bo_ (Table 2).

**TABLE 2:** Results of Mann–Whitney tests for the comparison of δ^18^O_bo_ and δ^13^C_bo−de_ between sexes (a) and funerary treatment (b–d)

(a)	Females				Males				
	*n*	Mean	Median	SD	*n*	Mean	Median	SD	*p* (Mann–Whitney *U* test)
δ^18^O_bo_	17	15.0	15.1	0.5	23	14.8	14.8	0.7	0.29
Δ^13^C_bo−de_ ^a^	12	−0.7	0.5	2.9	14	0.8	0.7	2.6	0.33

(b)	Supine				Prone or on the side				
	*n*	Mean	Median	SD	*n*	Mean	Median	SD	*p* (Mann–Whitney *U* test)
δ^18^O_bo_	39	14.7	14.8	0.8	10	14.9	15.0	0.8	0.71
Δ^13^C_bo−de_ ^a^	22	0.0	0.4	3.0	4	0.8	0.8	0.6	0.64

(c)	Without grave goods				With grave goods				
	*n*	Mean	Median	SD	*n*	Mean	Median	SD	*p* (Mann–Whitney *U* test)
δ^18^O_bo_	9	14.3	14.7	1.2	40	14.8	14.9	0.7	0.18
Δ^13^C_bo−de_ ^a^	2	−2.6	−2.6	4.2	24	0.3	0.7	2.7	0.21

(d)	Burial without structure				Burial with structure				
	*n*	Mean	Median	SD	*n*	Mean	Median	SD	*p* (Mann–Whitney *U* test)
δ^18^O_bo_	42	14.7	14.8	0.8	7	15.0	15.1	0.7	0.50
Δ^13^C_bo−de_ ^a^	21	0.2	0.8	2.0	5	−0.4	0.3	5.4	0.31

*Note*: n: number of individuals; SD: standard deviation. Data on δ^13^C_bo−de_ are available only for adults.

^a^ Only adult individuals.

Finally, the comparison of *δ* ^13^C_de_ from the two teeth of the “funerary/paleopathological outlier” US2807 highlights a minimal shift (0.2‰) from URM2 (−20.1‰V‐PDB) to LLP4 (−19.9‰V‐PDB).

## 4 DISCUSSION

Before discussing our results, it is worth mentioning some issues affecting our analyses. These relate to the size and demographic composition of our sample, and the analyzed elements and tissues.

Our sample size is admittedly small, and this becomes important when evaluating the power of our statistical analyses. This cautionary note seems particularly important when considering the sex bias in our sample (17 females and 23 males). Having larger subsamples of sexed individuals would allow a more solid exploration of the possible links between mobility, sex, and gender. Finally, the inclusion of other stable isotope elements and different skeletal tissues (e.g., *δ* ^18^O and ^87^Sr/^86^Sr from tooth enamel, *δ* ^34^S from bone collagen) may allow refining the picture emerged from this study.

### 4.1 What is the frequency of potential nonlocals at SV, and what does it tell about the type of human movements in this population?

Oxygen isotopic values at SV show a low variability and indicate a source of drinking water featuring relatively constant and homogeneous isotopic characteristics.

Based on the position of the site, and the water isotopic data, it is plausible to identify this source with the Adige river. This, in turn, suggest that most of the individuals spent their adult life in the surrounding of SV. The only individual showing a clearly different *δ* ^18^O_bo_ value is US 3244A. This 3–6‐months‐old nonadult was likely breastfed at the time of death (as further suggested by the elevated nitrogen isotopic ratio) (also see Laffranchi et al., 2018). On the other end, the breastfeeding effect seems not to be reflected in the observed *δ* ^18^O_bo_ value (Britton et al., 2015) which is the lowest one in the analyzed sample. The observed *δ* ^18^O_bo_ value would therefore reflect that of the mother. Either this infant was born “locally” (i.e., in the surrounding of SV) from a nonlocal woman, or both arrived at SV from somewhere else. Although indicating a specific geographic location based on isotopic values is notoriously difficult (cf. Lightfoot & O'Connell, 2016; Milella et al., 2019), some insight may be obtained by comparing calculated *δ* ^18^O_dw_ with modern data from rivers, springs, and rainwater. The conversion of *δ* ^18^O_ph_ to *δ* ^18^O_dw_ for US3244A results in a value of −16‰ V‐SMOW (Table S1), which indicates a higher altitude (mountain) environment, versus a mean value for the rest of the burial community of −10.9 ± 1‰ V‐SMOW. The latter range is consistent with local waters (Figure S2) and published *δ* ^18^O values for the Adige river in the area of Verona (average: −11.9 ± 0.6 considering the measurements from Parona, 6 km north from SV– Natali et al., 2016), although slightly different from rainfall *δ* ^18^O values for the same area (−7.7‰ ± 2‰—Longinelli & Selmo, 2003 and −7.1‰ ± 3.1‰—Bowen & Revenaugh, 2003; Bowen, 2021, http://www.waterisotopes.org). Giustini et al. (2016) highlight homogenous rainfall oxygen isotopic values (between −7‰ and −8‰) for the Northeastern Po plain (Verona: −7.65‰) (see figure 8 and supplementary data in Giustini et al., 2016). Conversely, the isotopic ratio of US3244A (and/or its mother we probably did not detect in our burial community) would point to a location featuring high altitude (Giustini et al., 2016; Natali et al., 2016). Particularly depleted isotopic values suggestive of mountain environments are also shown by US2603, 2807, 2890, and 3309 (three adult males and one infant of 2–3 months). The particularly negative *δ* ^18^O values of these samples allow excluding a diagenetic effect. Phosphate recrystallization in equilibrium with local meteoric waters (whose *δ* ^18^O ranges between −6 and −8—Giustini et al., 2016) and annual soil temperature of 15°C would make us expect higher or less negative values than those observed. These results further confirm therefore the good quality of our samples.

Regarding the location of these high‐altitude environments, the most likely hypothesis is that these individuals arrived at SV from a location in the Alpine area. Published *δ* ^18^O values for the upper stretch of the Adige river (elevation above 180 m) are indeed on average −12.9 ± 0.7 V‐SMOW, with the most depleted values found at sampling locations above 880 m (Figures 1c and S2b) (Natali et al., 2016). The inverse correlation between elevation and *δ* ^18^O is further demonstrated by our water sample from the upper stretch of the Adige (Bolzano, 262 m: −12.27‰, Figure S2a). Similarly, published rainfall *δ* ^18^O values show the more negative values in the Alpine area and, for isolated locations, the Central Apennines (Giustini et al., 2016). Compared with the alpine area the Apennines seem, however, an unlikely place of origin for US3244A, US2603, 2807, 2890, and 3309 based on the geographic location and the archeological features of the analyzed necropolis. First, SV is closer to the Alps, to which it is connected by a main waterway (Adige). Second, the available archeological data (still under study) point to a connection (at least cultural) between SV and transalpine areas. This is for example suggested by the presence of pins of transalpine typologies and ceramics carrying Rhaetian Lepontian inscriptions (Marchesini & Stifter, 2018; Solinas, 2015).

Another male (US3274: middle adult) presents the most enriched *δ* ^18^O_bo_ value (16.0 ‰). When combined with his depleted *δ* ^13^C_bo_ and *δ* ^13^C_de_ values this may cautiously suggest his origin from an area lacking rivers with high‐elevation recharge areas, that is, an area of plains, surrounded by mountains of low altitude (e.g., more southern regions—cf. Milella et al., 2019; Prowse et al., 2004). We want to stress that, given the large 3MAD range at SV, and the large error associated with the use of *δ* ^18^O_dw_ for indicating a geographic origin (Lightfoot & O'Connell, 2016) these hypotheses need to be considered with caution. Furthermore, our interpretation needs to take into account a series of factors, besides geographic origin, potentially influencing the observed *δ* ^18^O_bo_ values. These include food preparation techniques (e.g., cooking), consumption of imported foodstuff, physiological, and metabolic factors, such as body size, nutritional stress, disease, amount of water in the diet (Brettell et al., 2012; Lightfoot & O'Connell, 2016; Moreiras Reynaga et al., 2021; Pederzani & Britton, 2019; White et al., 2000).

As mentioned, the choice to (cautiously) consider *δ* ^13^C as a potential marker of mobility is suggested by the correlation between this variable and environmental factors. In the case of SV, the cultivation of millet, although possible only during spring–summer, resulted in a strong C_4_ signal in this population (Laffranchi, 2016; Laffranchi et al., 2016). The cultivation of millet in the Po plain is documented since the Bronze Age (Tafuri et al., 2009; Tafuri et al., 2018; Varalli et al., 2016) and continued to be practiced during Late Antiquity and early Middle Ages (Ganzarolli et al., 2018; Laffranchi, Mazzucchi, et al., 2020; Marinato, 2016, 2017, 2019; Maxwell, 2019). In contrast, the low minimum temperatures characterizing mountain areas make the latter incompatible with the cultivation of C_4_ plants (Teeri & Stowe, 1976). This is further confirmed by a recent isotopic analysis of Early medieval contexts from the Italian Alps showing a substantial reliance on C_3_ plants, and, in general, more depleted carbon isotopic ratios at higher altitudes (Paladin et al., 2020).

Clearly, *δ* ^13^C values per se cannot point unambiguously to an individual's nonlocal origin, since they may be related to personal dietary preferences and/or cultural customs. However, in those cases where both oxygen and carbon isotopic ratios fall outside (or on the edge) of the sample range, we may try to postulate some more explicit hypotheses. This is the case, for example, of two of the potential nonlocal males based on *δ* ^18^O_bo_ (US2807 and 2890), who also show some of the more depleted *δ* ^13^C_de_ values (for US 2807 also for *δ* ^13^C_bo_) in the sample. Both oxygen and carbon isotopic ratios would support therefore a nonlocal origin of these individuals.

The differences between carbon isotopic values from bone and dentine collagen deserve some further consideration. We recall that our aim in comparing these types of data was exploring intraindividual lifetime differences potentially related to changes in diet and/or mobility. A change in diet between infancy and late adulthood is indeed suggested for three males (US 905, 2890, and 2894) and three females (US 2528, 2685, and 3259). Of these six cases, three females' and two males' ∆^13^C_bo−de_ values are consistent with a shift toward a higher dietary contribution of C_4_ plants. Previous analyses of SV highlighted the important dietary contribution of C_4_ plants (likely millet) in this population (see Laffranchi et al., 2016). Furthermore, all inhumations at SV have shown a certain variability in *δ* ^13^C from bone collagen with an absolute mean difference of minimal 0.4, and maximal 2.7 (see Laffranchi et al., 2018). In contrast, the ∆^13^C_bo−de_ values for the five individuals discussed here show an absolute difference of minimum 3 and maximum 8. Considering these data, the depleted dentine values of these individuals may be related to them having spent their infancy in a location featuring less availability of C_4_ plants followed by their movement (at least during the last years before death) to SV. A lack of relocation during the last years before death is also suggested by the fact that ∆^13^C_bo−de_ outliers tend to fall in the *δ* ^18^O range of the population. The discussion of the isotopic values of the middle adult male US2894 with a largely positive ∆^13^C_bo−de_ (depleted bone isotopic value) is more difficult. This result may be related to a specific diet during infancy (e.g., during the weaning process) including a high contribution of C_4_ plant products, followed by an adult diet more based on C_3_ resources. We cannot establish if this was due to dietary preferences, high consumption of proteins from animals feeding on C_3_ plants (note that the same individual also shows the highest nitrogen isotopic value among adults at SV), a recent movement to SV from somewhere else, or to a combination of these factors.

Boiling down these considerations to actual numbers, and applying a parsimonious approach, the frequency of nonlocals would be somewhere between 1/49 (2%) and 6/49 (12%). Comparative data for continental European Late Iron Age contexts are available for Nebringen (Germany, 400–250 BCE), the Glauberg (Germany, ca. 400 BCE), Monte Bibele (Italy, 5th–3rd c. BCE), Monterenzio Vecchio (Italy, 4th–3rd c. BCE), Münsingen Rain (Switzerland, ca. 450–150 BCE), Basel Gasfabrik (Switzerland, 200–80 BCE), Radovesice I, Radovesice II and Kutná Hora (Bohemia, 380–250 BCE) (Hauschild et al., 2013; Knipper et al., 2014, 2018; Moghaddam et al., 2016; Scheeres et al., 2013, 2014; Sorrentino et al., 2018). It should be noted however that none of these studies used the same approach employed here. Rather, they usually include the analysis of strontium and oxygen isotopic ratios from dental enamel, and/ or sulfur from bone collagen. The criteria used to define local isotopic ranges also differ from those employed in our study (3MAD). All these considerations make it clear that we can only loosely compare our results with those from these studies, and just in terms of the relative numerosity of nonlocals. Frequencies of nonlocals range from a minimum of ca. 11% (4/35) in Radovesice (Scheeres et al., 2014) to higher frequencies like the 42% (8/19) at the princely seat of Glauberg (Knipper et al., 2014) and 57% (13/23) at Monterenzio Vecchio (Sorrentino et al., 2018). SV would therefore lie in the lower range of these percentages, a result for which we can propose various, and not mutually exclusive explanations. The aforementioned methodological differences are important and likely to influence the relative frequency of isotopic outliers in a sample. Differences between the demographic and the economic composition of analyzed population may also play a role, especially if the sex, age, and/or social standing and profession of an individual made them more (or less) likely to move. The type of network each population is part of (economic, cultural, etc.) is also likely to influence the degree of mobility of its members. We also need to question the reliability of our analytical approach in capturing various scales of human movement. In particular, an important point to consider is the difference between mobility and migration (Kern, 2012;Nehlich et al., 2009; Tilly, 1978), two terms that are often used as synonyms although they indicate different phenomena. We consider mobility processes those involving regional and periodic moves in one's home region, whereas migrations would include discrete relocation over long distances (Nehlich et al., 2009; Tilly, 1978). Climatic similarities make it possible that a large part of short‐distance mobility at SV was left undetected by our analyses, which would be more sensitive to long‐distance movements. The picture emerging from this study would therefore be an underestimation of the actual frequency of mobility in the analyzed population.

As mentioned when discussing the limitation of our study, we plan to extend our analyses by including other elements (strontium, sulfur) and *δ* ^18^O from dental enamel. Besides providing a better basis for comparison with other studies, these data would allow more insights about the types of mobility in this context.

### 4.2 Is there any pattern among assumed nonlocals based on sex and/or age?

At both Nebringen and Monte Bibele nonlocals or mobile individuals were mainly males (Scheeres et al., 2013), and a similar conclusion was reached for Radovesice and Kutná Hora (Scheeres et al., 2014). For Münsigen Rain (Hauschild et al., 2013; Moghaddam et al., 2016) the frequency of nonlocals is relatively low (14.7% according to Hauschild et al., 2013) with an equal distribution between the sexes. Conversely, data from the Glauberg and Basel Gasfabrik highlight a higher female mobility (Knipper et al., 2014, 2018). At SV, the four adults with *δ* ^18^O_bo_ values deviating from the rest of the burial community are males, whereas the six individuals showing large deviations between dentine and bone collagen include an equal number of both sexes (three males and three females). Taken together, these results suggest a slightly higher mobility for males. Our previous works on entheseal changes (Laffranchi, Charisi, et al., 2020) at SV pointed at differences between sexes in physical activity. Males appear to have experienced higher levels of biomechanical stress than females, likely due to the presence in this society of a sex subdivision of labor. The involvement of males in different professions and activities may in turn have influenced their relative likelihood to move.

### 4.3 Is there an association between the isotopic signature of postulated nonlocality and the type of funerary treatment (body position, presence of grave goods)?

Our analyses did not highlight any association between isotopic data and the three selected funerary variables. This in turn suggests a lack of association between geographic origin and funerary treatment. Our result is not surprising since social status is unlikely to follow a simplistic dichotomy between “locals” and “nonlocals.” Additional factors, such as specific geographic origin, the timing of movement, kinship relationship, in conjunction with processes of cultural integration are likely to lead to complex, fluid, and heterogeneous scenarios (Eckardt, 2010; Laffranchi et al., 2019; Levitt, 2018; Milella et al., 2019).

The unsuitability of simplistic categories and the need for nuanced interpretation are well exemplified by the “funerary outlier male” US2807. The isotopic ratios from bones and teeth depict in this case a diachronic enrichment in *δ* ^13^C. Furthermore, he stands out for presenting the most depleted *δ* ^13^C_bo_ at SV, and one of the more depleted *δ* ^18^O_bo_. The preliminary archeological interpretation of the wooden container associated with this burial is of a small tub or barrel, an object undocumented so far in other necropolises of La Tène chronology in the Italian peninsula (Laffranchi et al., 2015). According to ancient Roman and Greek sources (Pliny, Strabo, and Caesar), the wooden barrel was a container largely used among “Celtic” populations (e.g., for storing wine—Plin., Nat. Hist. XIV, 132). Interestingly, archeological remains of barrels have been found along the courses of the rivers Rhine, Danube, and Rhone, in Switzerland, along the French Mediterranean coast, and in Great Britain, and all date back to the 1st–3rd century AD (Baratta, 1994). The wooden object associated with the middle adult male (US 2807) may therefore represent not only a *unicum* for the Italian peninsula but also one of the earliest documented barrels in Continental Europe. Further typological and archeological analyses are needed to corroborate this preliminary hypothesis, and the study of this particular object as well as the grave goods typology of SV is ongoing (Salzani, Personal communication).

Comparing this information with the isotopic and radiocarbon data, it is tempting to propose a specific “residential history” of this individual. It is possible that the male (US2807) originated from a high altitude, possibly Alpine region (*δ* ^18^O_bo_ and *δ* ^13^C data), and was among the first “Celtic” settlers in the area of Verona (^14^C data), where he died relatively shortly after his arrival (intraskeletal and intertooth *δ* ^13^C data; ∆^13^C_bo−de_ data). Naturally, although suggestive, this is only a preliminary hypothesis that will have to be explored more extensively through biogeochemical and biomolecular analyses (additional isotopic analyses, additional radiocarbon dating, and ancient DNA), and by more detailed scrutiny of the different typological phases of the necropolis.

## 5 CONCLUSIONS

In this study, we investigated the presence of nonlocals at Seminario Vescovile (Northeastern Italy, 3rd–1st century BCE) by means of an analysis of δ^13^C from bone and dentine collagen and δ^18^O from bone phosphate. Results suggest a nonlocal origin for one individual, and possibly for other five, with no significant pattern according to sex or funerary treatment. With one exception, nonlocals appear to have moved to SV from a higher altitude, possibly an alpine environment. Overall, our results depict a population featuring reduced levels of residential mobility or mostly short‐distance movements. At the same time, the possible alpine origin of the nonlocal individuals confirms expectations based on historical sources and archeological data about the pre‐Roman populations of Northern Italy. This study is the first to explore residential mobility in a “Celtic” population in the northeast of the Italian peninsula, a crucial area for the cultural and demic exchanges between Mediterranean and central European territories. Accordingly, our data contribute to a better understanding of the heterogeneous processes shaping the cultural and biological variability of the Italian peninsula during the last centuries BCE.

This research opens various research questions. These include (a) what kind of movements (long‐vs. short distance mobility, frequency of nonlocals, sex and age patterns) characterized the *entire* burial community of SV? (b) What genetic variation characterized SV, and what genetic relationships linked this population with other, transalpine “Celtic” groups? (c) What kind of relationship linked mobility, kinship patterns, and social organization in this context, and how do these patterns compare with other, transalpine populations? Planned isotopic, aDNA, and archeological analyses will provide the opportunity to address these questions.

## CONFLICT OF INTEREST

The authors declare that they have no conflict of interest.

## AUTHOR CONTRIBUTIONS

**Zita Laffranchi:** Conceptualization (lead); data curation (lead); formal analysis (equal); funding acquisition (equal); investigation (lead); methodology (equal); project administration (equal); resources (equal); validation (equal); visualization (equal); writing – original draft (lead); writing – review and editing (lead). **Arsenio Granados‐Torres:** Conceptualization (supporting); data curation (equal); formal analysis (supporting); investigation (supporting); methodology (equal); validation (supporting); writing – review and editing (supporting). **Sandra Lösch:** Conceptualization (supporting); funding acquisition (supporting); investigation (supporting); methodology (supporting); writing – review and editing (equal). **Albert Zink:** Conceptualization (supporting); funding acquisition (lead); project administration (lead); supervision (equal); writing – review and editing (supporting). **Irene Dori:** Writing – review and editing (supporting). **Antonio Delgado‐Huertas:** Conceptualization (equal); data curation (equal); funding acquisition (lead); investigation (equal); methodology (lead); project administration (equal); resources (lead); software (equal); supervision (equal); validation (equal); writing – original draft (equal); writing – review and editing (equal). **Marco Milella:** Conceptualization (equal); data curation (equal); formal analysis (lead); funding acquisition (lead); investigation (lead); methodology (lead); project administration (lead); resources (equal); software (equal); supervision (lead); validation (equal); visualization (lead); writing – original draft (lead); writing – review and editing (lead).

## Supporting information


**Figure S1** (a) X‐ray diffraction (XRD) patterns of US3274, 2807, 3309, 3243, and 3244A compared with those from a well‐crystallized apatite standard (COD 9010052: bottom, black curve), and from a sediment from Mexico rich in phosphates (PMF‐1: red curve). The latter serves as reference for a material that has already undergone diagenesis. Our samples show the typical broad bands, without sharp peaks, of a more amorphous material that has not yet recrystallized due to the effects of diagenesis. US3274 and US3244A are the samples showing the two extremes of *δ* ^18^O values (maximum and minimum, respectively). The dotted square highlights the phosphate area in the plot. (b) Oxygen isotopic ratios of the analyzed samples (red diamonds).

**Figure S2** (a) Converted *δ* ^18^O_dw_ from SV compared with oxygen isotopic ratios from the water samples collected for this study (1–10) and with published values for the Adige River and meteoric water in the area of interest. Numbers identify our water sampling locations: see Table S3 for details. The blue star highlights the only sample from an area not surrounding SV (Bolzano–Upper course Adige River); (b) Converted *δ* ^18^O_dw_ from SV compared with the calculated averages of published values at different elevations along the course of the Adige River (original data from Natali et al., 2016).

**Table S1** Complete dataset used in the study. y: years; M: male; F: female; NA: sex undetermined. Tooth samples are indicated by jaw (upper—U, lower—L), side (left—L, right—R), tooth type (incisor—I, Molar—M, Premolar—P) and tooth order (first—1,second—2, third—3).
The identification of premolars follows the criterion: P3: first premolar; P4: second premolar.
*: US2807 was sampled twice for bone and dentine collagen

**Table S2** Quality criteria for all analyzed collagen samples.

**Table S3** *δ*D ‰ and *δ* ^18^O of the water samples collected for this study.

**Table S4** Comparison of *δ* ^13^C from all bone and dentine samples. n: number of samples; min: minimum value; max: maximum value; SD: standard deviation

## Data Availability

The data that supports the findings of this study are available in the supplementary material of this article.
