# Supplementary figures and images for: “Celts” up and down the Alps. Insights on mobility patterns in the pre‐Roman/Celtic population from Verona (NE Italy, 3rd–1st c. BCE): A multi‐isotopic approach
Source: Am J Biol Anthropol. 2022 Apr 26;178(3):513–29. doi: 10.1002/ajpa.24523 (PMC9544713; doi:10.1002/ajpa.24523)

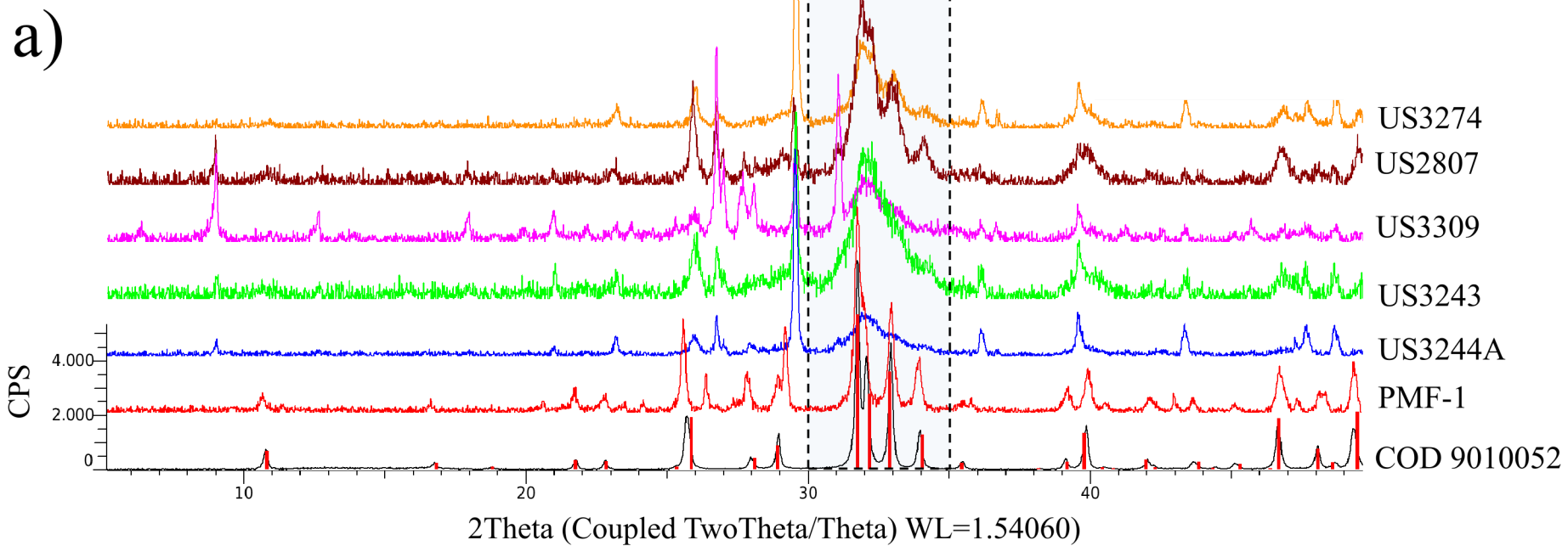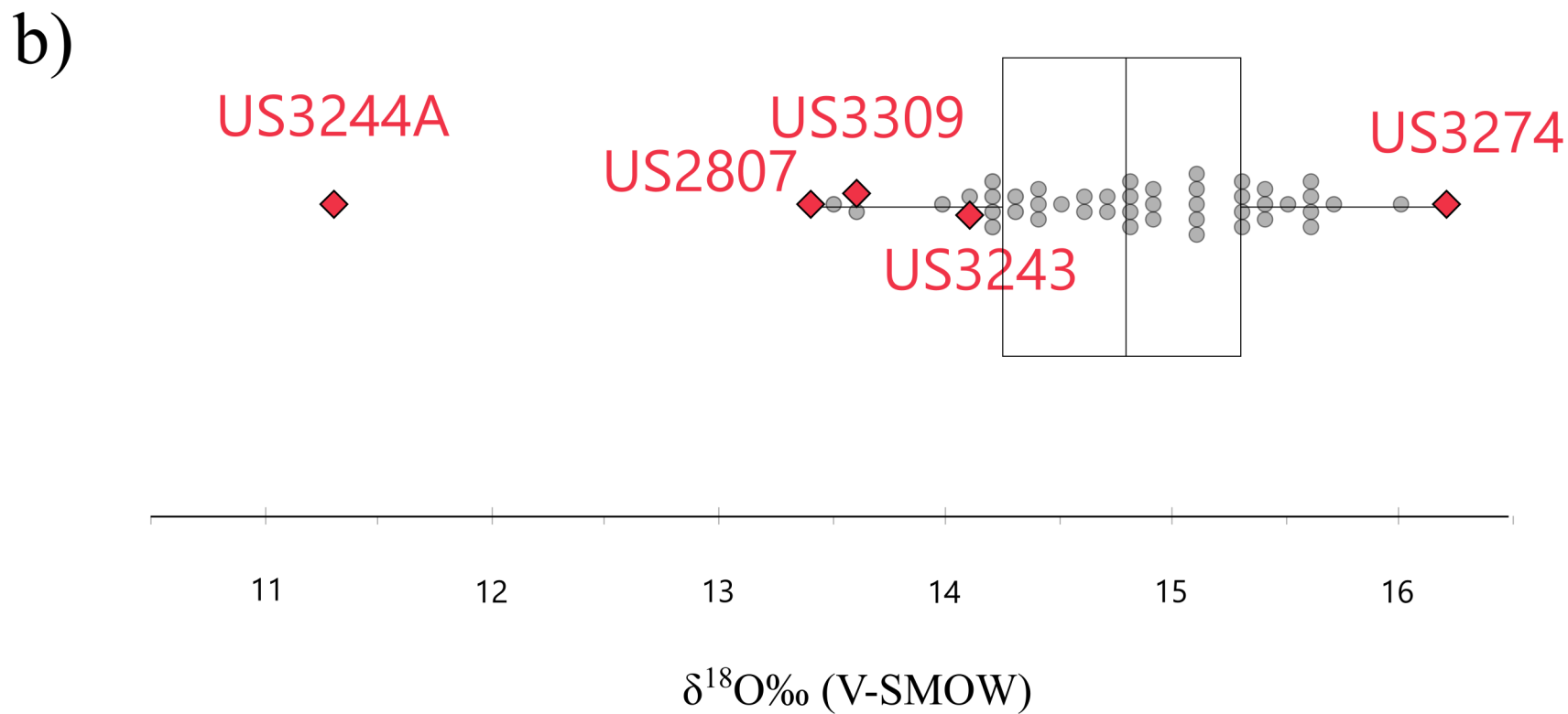

Supplement: Supplementary file 1 — Figure S1 (a) X‐ray diffraction (XRD) patterns of US3274, 2807, 3309, 3243, and 3244A compared with those from a well‐crystallized apatite standard (COD 9010052: bottom, black curve), and from a sediment from Mexico rich in phosphates (PMF‐1: red curve). The latter serves as reference for a material that has already undergone diagenesis. Our samples show the typical broad bands, without sharp peaks, of a more amorphous material that has not yet recrystallized due to the effects of diagenesis. US3274 and US3244A are the samples showing the two extremes of δ 18O values (maximum and minimum, respectively). The dotted square highlights the phosphate area in the plot. (b) Oxygen isotopic ratios of the analyzed samples (red diamonds). [file AJPA-178-513-s006.pdf]

a)

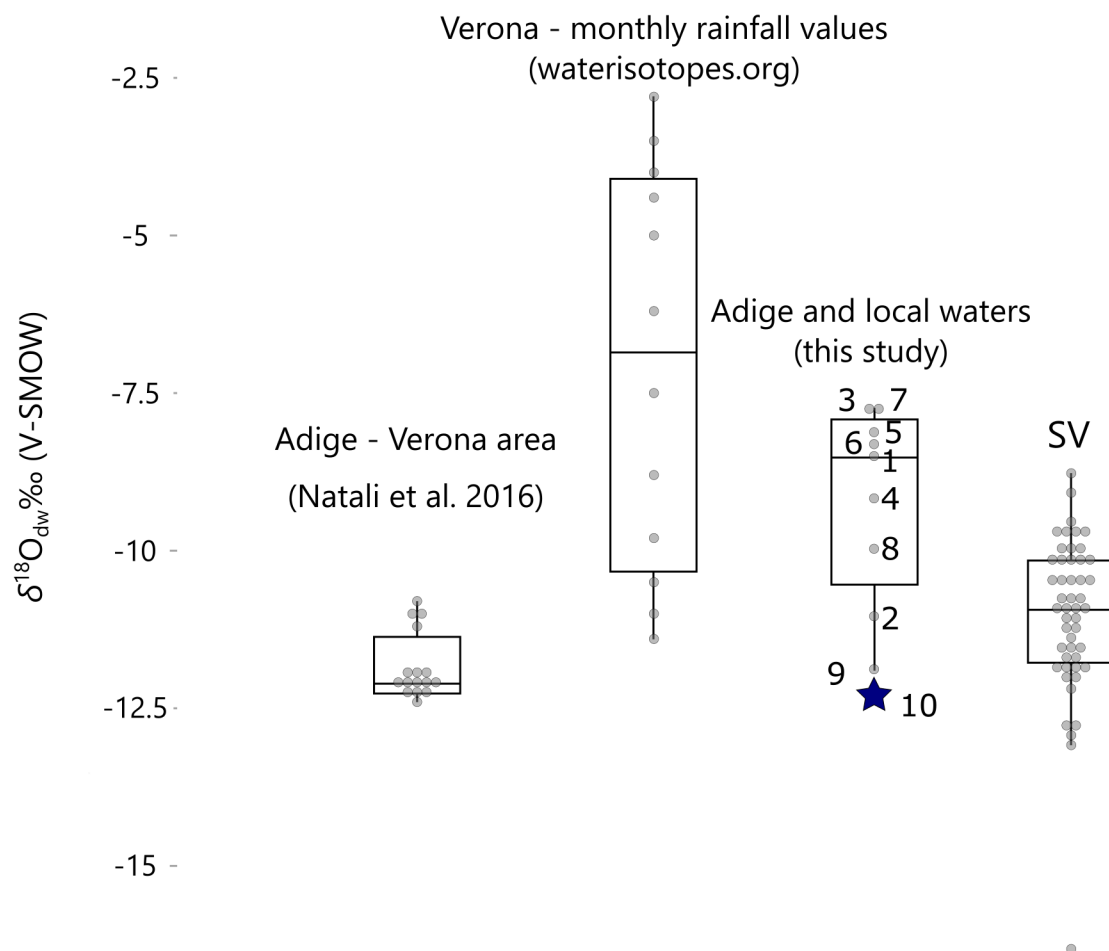

b)

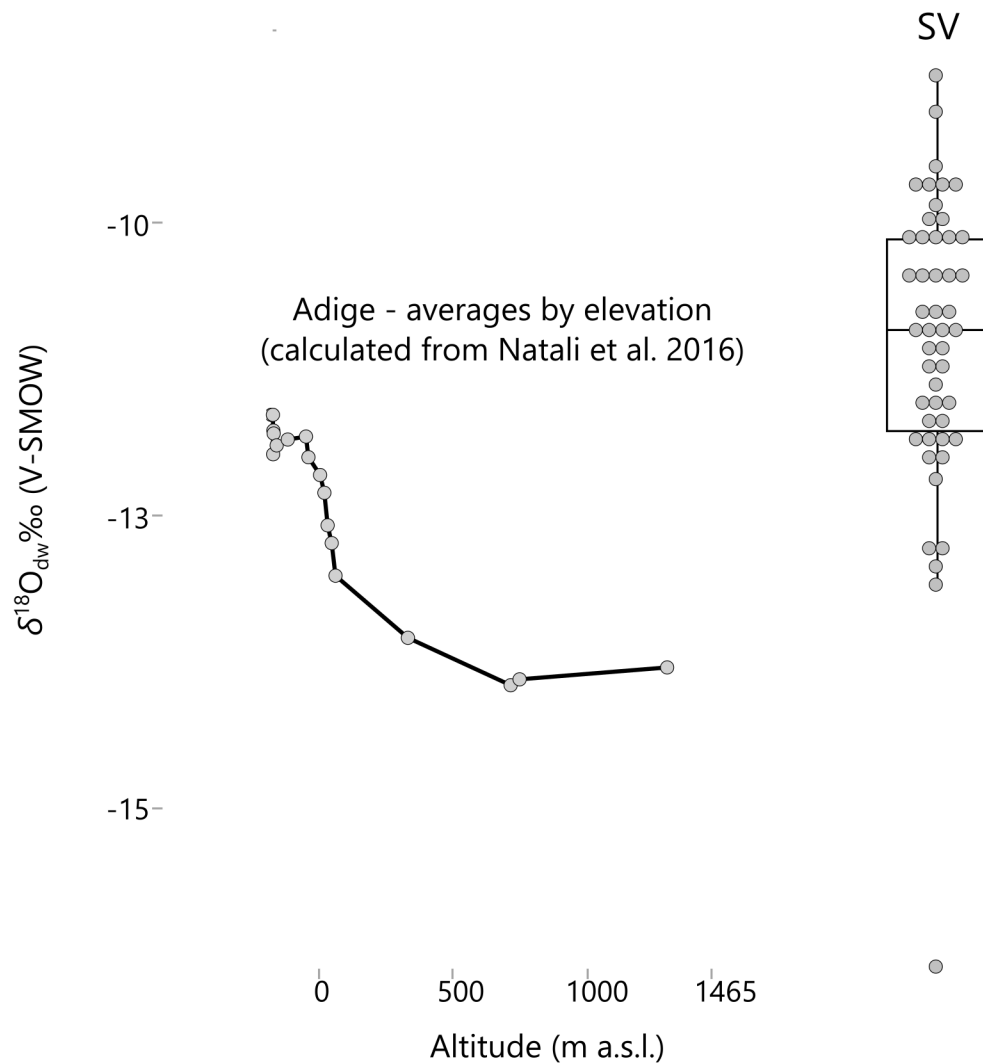

Supplement: Supplementary file 2 — Figure S2 (a) Converted δ 18O dw from SV compared with oxygen isotopic ratios from the water samples collected for this study (1–10) and with published values for the Adige River and meteoric water in the area of interest. Numbers identify our water sampling locations: see Table S3 for details. The blue star highlights the only sample from an area not surrounding SV (Bolzano–Upper course Adige River); (b) Converted δ 18O dw from SV compared with the calculated averages of published values at different elevations along the course of the Adige River (original data from Natali et al., 2016). [file AJPA-178-513-s005.pdf]
